# Correction to Abrams et al. (2015)

**DOI:** 10.1037/pac0000115

**Published:** 2015-04-20

In the article “Equality Hypocrisy, Inconsistency, and Prejudice: The Unequal Application of the Universal Human Right to Equality,” by Dominic Abrams, Diane M. Houston, Julie Van de Vyver, and Milica Vasiljevic (*Peace and Conflict: Journal of Peace Psychology*, Vol. 21, No. 1, pp. 28–46. http://dx.doi.org/10.1037/pac0000084), the copyright should have been “© 2015 The Author(s)”. The author note also should have included the following license statement, “This article has been published under the terms of the Creative Commons Attribution License (CC-BY, http://creativecommons.org/licenses/by/3.0/), which permits unrestricted use, distribution, and reproduction in any medium, provided the original author and source are credited. Copyright for this article is retained by the author(s). Author(s) grant(s) the American Psychological Association the exclusive right to publish the article and identify itself as the original publisher.” The online version of this article has been corrected.

